# Mitochondrial Contact Site and Cristae Organization System and F_1_F_O_-ATP Synthase Crosstalk Is a Fundamental Property of Mitochondrial Cristae

**DOI:** 10.1128/mSphere.00327-21

**Published:** 2021-06-16

**Authors:** Lawrence Rudy Cadena, Ondřej Gahura, Brian Panicucci, Alena Zíková, Hassan Hashimi

**Affiliations:** a Institute of Parasitology, Biology Center, Czech Academy of Sciences, České Budějovice, Czech Republic; b Faculty of Science, University of South Bohemia, České Budějovice, Czech Republic; University at Buffalo

**Keywords:** ATP synthase, MICOS, *Trypanosoma*, evolution, mitochondria

## Abstract

Mitochondrial cristae are polymorphic invaginations of the inner membrane that are the fabric of cellular respiration. Both the mitochondrial contact site and cristae organization system (MICOS) and the F_1_F_O_-ATP synthase are vital for sculpting cristae by opposing membrane-bending forces. While MICOS promotes negative curvature at crista junctions, dimeric F_1_F_O_-ATP synthase is crucial for positive curvature at crista rims. Crosstalk between these two complexes has been observed in baker’s yeast, the model organism of the Opisthokonta supergroup. Here, we report that this property is conserved in Trypanosoma brucei, a member of the Discoba clade that separated from the Opisthokonta ∼2 billion years ago. Specifically, one of the paralogs of the core MICOS subunit Mic10 interacts with dimeric F_1_F_O_-ATP synthase, whereas the other core Mic60 subunit has a counteractive effect on F_1_F_O_-ATP synthase oligomerization. This is evocative of the nature of MICOS–F_1_F_O_-ATP synthase crosstalk in yeast, which is remarkable given the diversification that these two complexes have undergone during almost 2 eons of independent evolution. Furthermore, we identified a highly diverged, putative homolog of subunit e, which is essential for the stability of F_1_F_O_-ATP synthase dimers in yeast. Just like subunit e, it is preferentially associated with dimers and interacts with Mic10, and its silencing results in severe defects to cristae and the disintegration of F_1_F_O_-ATP synthase dimers. Our findings indicate that crosstalk between MICOS and dimeric F_1_F_O_-ATP synthase is a fundamental property impacting crista shape throughout eukaryotes.

**IMPORTANCE** Mitochondria have undergone profound diversification in separate lineages that have radiated since the last common ancestor of eukaryotes some eons ago. Most eukaryotes are unicellular protists, including etiological agents of infectious diseases, like Trypanosoma brucei. Thus, the study of a broad range of protists can reveal fundamental features shared by all eukaryotes and lineage-specific innovations. Here, we report that two different protein complexes, MICOS and F_1_F_O_-ATP synthase, known to affect mitochondrial architecture, undergo crosstalk in T. brucei, just as in baker’s yeast. This is remarkable considering that these complexes have otherwise undergone many changes during their almost 2 billion years of independent evolution. Thus, this crosstalk is a fundamental property needed to maintain proper mitochondrial structure even if the constituent players considerably diverged.

## INTRODUCTION

Mitochondria are ubiquitous organelles that play a central role in cellular respiration in aerobic eukaryotes alongside other essential processes, some of which are retained in anaerobes (1). These organelles have a complex internal organization. While the mitochondrial outer membrane is smooth, the inner membrane is markedly folded into invaginations called cristae, the morphological hallmark of the organelle in facultative and obligate aerobes (2, 3). Cristae are enriched with respiratory chain complexes that perform oxidative phosphorylation (4, 5), and eukaryotes that cannot form these complexes lack these ultrastructures (1, 6).

Mitochondrial morphology differs among species, tissues, and metabolic states. Nevertheless, these variations are derived from common structural features, some of which were likely inherited from an endosymbiotic alphaproteobacterium that gave rise to the organelle (1, 7, 8). Cristae contribute to this variety as they can assume different shapes, such as platelike lamellar cristae, which decorate yeast and animal mitochondria, and the paddlelike discoidal cristae seen in discoban protists (9). These shapes are at least in part determined by protein complexes embedded within the crista membranes.

The mitochondrial contact site and cristae organization system (MICOS) is a heterooligomeric protein complex that is responsible for the formation and maintenance of crista junctions, narrow points of attachment of cristae to the rest of the inner membrane (10, 11). Crista junctions serve as diffusion barriers into and out of cristae, as these structures cease to act as autonomous bioenergetic units upon MICOS ablation (12). The two core MICOS subunits Mic10 and Mic60, both of which are well conserved throughout eukaryotes (13, 14), have demonstrated membrane modeling activity that contributes to constriction at crista junctions (15–18).

The F_1_F_O_-ATP synthase (here “ATP synthase”) dimers also influence the shape of cristae (19). Throughout eukaryotes, dimeric ATP synthase assembles into rows or other oligomeric configurations that promote positive curvature at crista rims (20–22). In yeast and animals, both belonging to the eukaryotic supergroup Opisthokonta (23), ATP synthase dimerization depends on membrane-embedded F_O_ moiety subunits e and g (24–26). Although these subunits do not directly contribute to intermonomer contacts, deletion of either of them hinders dimer formation (20) and subsequently results in the emergence of defective cristae (26, 27).

Crosstalk between the crista-shaping factors MICOS and ATP synthase has been demonstrated in yeast. A fraction of Mic10 physically interacts with ATP synthase dimers, presumably via subunit e, and the overexpression of Mic10 leads to the stabilization of ATP synthase oligomers (28, 29). An even more pronounced accumulation of ATP synthase oligomers was observed upon the deletion of the Mic60 gene, but no physical interaction between Mic60 and any ATP synthase subunits has been reported (30). This interplay between MICOS and ATP synthase is a remarkable and still unexplained phenomenon, as both complexes play critical yet apparently antithetical roles in shaping the inner membrane.

Here, we ask whether crosstalk between MICOS and ATP synthase dimers is a fundamental property of cristae. We employed the protist Trypanosoma brucei, a model organism that is part of the clade Discoba, which diverged from the Opisthokonta ∼1.8 billion years ago (23, 31). Because of their extended independent evolution, discoban MICOS (31–33) and ATP synthase (22, 34–36) differ radically from their opisthokont counterparts. Discoban MICOS has two Mic10 paralogs and an unconventional Mic60 that lacks the C-terminal mitofilin domain characteristic of other Mic60 orthologs (13, 14). Furthermore, unlike opisthokont MICOS, which is organized into two integral membrane subcomplexes, each assembled around a single core subunit, trypanosome MICOS is composed of one integral and one peripheral subcomplex. Discoban ATP synthase exhibits type IV dimer architecture, different from the canonical type I dimers found in opisthokonts (20–32), and has a dissimilar F_O_ moiety, which lacks obvious homologs of subunit e or g (37).

## RESULTS

### Depletion of trypanosome Mic60 affects oligomerization of F_1_F_O_-ATP synthase independent of other MICOS proteins.

**FIG 1:**
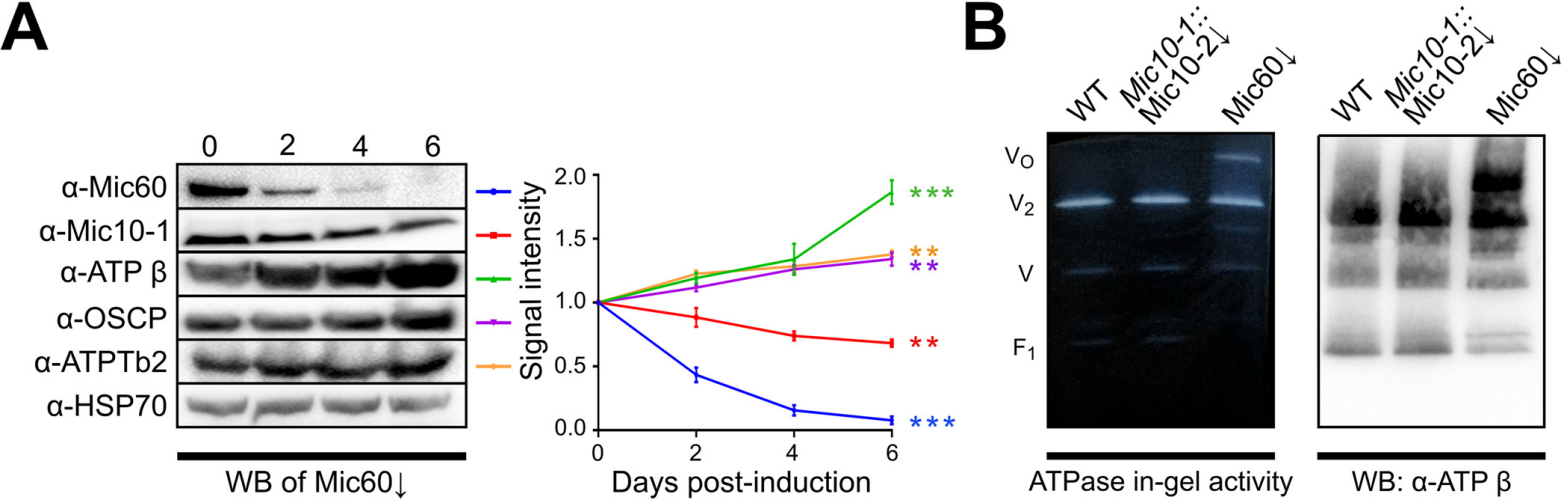
F_O_F_1_-ATP synthase oligomers are stabilized upon Mic60 depletion. (A) Representative immunoblots of whole-cell lysates from Mic60↓ cells over a 6-day course of RNAi induction with antibodies indicated on the left and densiometric quantification of immunoblots done in triplicate, with the signal normalized to unaffected HSP70 (error bars indicate standard deviations [SD]). Days postinduction are shown above the immunoblots. Line colors to the right of each immunoblot label the antibody signal intensities shown in the graph. The signal intensity is plotted in arbitrary units. The statistical significance of differences in signal intensities between 0 and 6 days after RNAi induction (i.e., the overall change in protein levels over the time course) is shown by asterisks on the right (**, *P* ≤ 0.01; ***, *P* ≤ 0.001). (B) Blue native PAGE using 1.5% DDM-solubilized mitochondria from Δ*Mic10-1* Mic10-2↓ and Mic60↓ RNAi cells at 5 days postinduction compared to the wild type (WT). ATP hydrolysis in-gel activity staining is shown on the left, and an immunoblot (WB) probed with anti-ATP synthase β-subunit antibody is shown on the right. V_O_, oligomer; V_2_, dimer; V, monomer; F_1_, free F_1_ moiety.

In T. brucei, the ablation of conserved MICOS components, Mic60 or both Mic10 paralogs simultaneously, resulted in impaired submitochondrial morphology characterized by elongated cristae adopting arc-like structures (32). Because in budding yeast, the knockout of Mic60 affects the oligomerization of ATP synthase (27), a major contributor to crista organization, we asked whether ATP synthase plays a role in the changes in mitochondrial ultrastructure observed after Mic10 and Mic60 depletion. After inducible RNA interference (RNAi) silencing of Mic60 (Mic60↓), we observed increased levels of ATP synthase subunits β, a component of the catalytic F_1_ sector; oligomycin sensitivity conferral protein (OSCP), a subunit of the peripheral stalk; and ATPTb2, a distant homolog of opisthokont subunit d. Simultaneously, levels of Mic10-1 were mildly reduced (Fig. 1A). Notably, RNAi induction of Mic10-2 in the Mic10-1 knockout cell line (Δ*Mic10-1* Mic10-2↓) resulted in the same crista defects as those in Mic60↓ cells (31) but did not result in detectable changes in steady-state levels of ATP synthase subunits (see Fig. S1A in the supplemental material). Thus, the accumulation of ATP synthase subunits documented upon Mic60 knockdown cannot be explained as a general consequence of morphologically elongated and detached cristae.

10.1128/mSphere.00327-21.1 FIG S1 Depletion of other MICOS subunits does not affect F_O_F_1_-ATP synthase oligomerization (Fig. 1). (A) Immunoblot of Δ*Mic10-1* Mic10-2↓ with antibodies indicated on the right. The wild type (WT) and days postinduction are shown at the top. (B) BN-PAGE of 1.5% *n*-dodecyl-β-d-maltoside-solubilized mitochondria from Mic20↓ and Mic32↓ cells at 5 days postinduction compared to the WT. ATP hydrolysis in-gel activity staining is shown on the left, and an immunoblot (WB) against anti-ATP β is shown on the right. V_2_, dimer; V, monomer; F_1_, free F_1_ moiety. Download FIG S1, TIF file, 2.3 MB. Copyright © 2021 Cadena et al. 2021 Cadena et al. https://creativecommons.org/licenses/by/4.0/ This content is distributed under the terms of the Creative Commons Attribution 4.0 International license.

The increased abundance of ATP synthase subunits prompted us to investigate if the depletion of Mic60 affects the oligomeric state of ATP synthase by immunodetection of ATP synthase complexes in 1.5% *n*-dodecyl-β-d-maltoside (DDM)-solubilized mitochondria resolved by blue native polyacrylamide gel electrophoresis (BN-PAGE) (Fig. 1B). RNAi induction in Mic60↓ but not in Δ*Mic10-1* Mic10-2↓ cells led to the stabilization of ATP synthase oligomers, which are labile to nonionic detergents in wild-type (WT) cells (38, 39). ATP hydrolysis in-gel activity staining demonstrated that the oligomers are enzymatically active. To confirm that the accumulation of detergent-resistant ATP synthase oligomers was exclusive to Mic60↓, we screened the effect of the depletion of two kinetoplastid-specific subunits of MICOS known to disrupt cristae, Mic32 and Mic20. Upon their depletion, no changes in ATP synthase oligomerization were observed on native gels (Fig. S1B). In conclusion, we present evidence that Mic60 depletion in T. brucei alters the oligomeric state of ATP synthase independently of the defects to cristae.

### Trypanosome Mic10-1 interacts with F_1_F_O_-ATP synthase.

**FIG 2:**
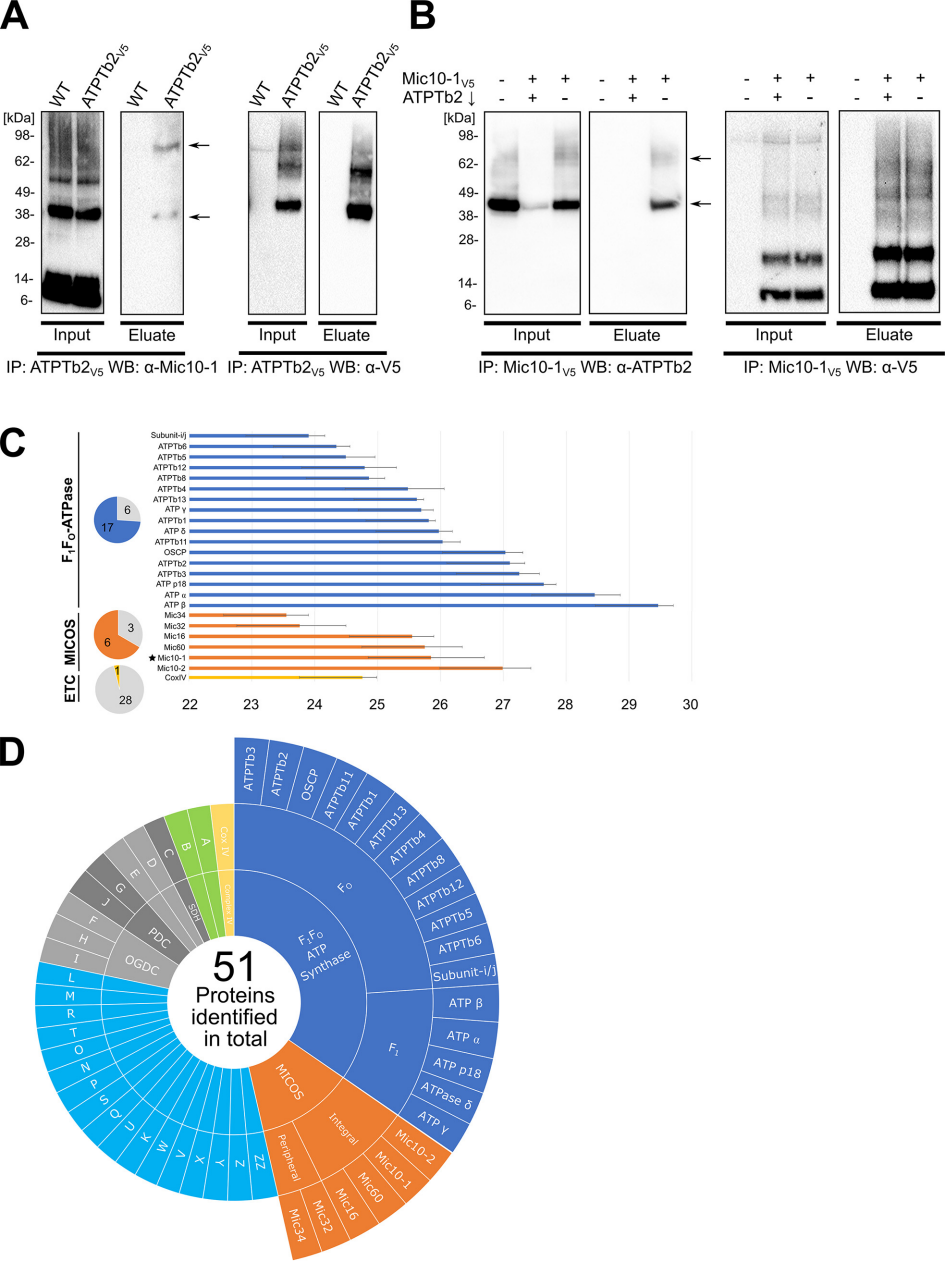
Mic10-1 interacts with F_O_F_1_-ATP synthase. (A) Immunoprecipitation (IP) with anti-V5 antibody of the wild type (WT) and ATPTb2-V5 from mitochondria cross-linked with 80 μM DSP. The input (10% of the eluate) and eluates were resolved by SDS-PAGE and immunoblotted (WB) with antibody against Mic10-1 and V5 peptide. Arrows indicate discernible bands corresponding to ∼35 kDa and ∼70 kDa. (B) Immunoprecipitation (IP) of WT, Mic10-1–V5:ATPTb2↓, and Mic10-1–V5 mitochondria cross-linked with 80 μM DSP. The input (10% of the eluate) and eluates were resolved by SDS-PAGE and immunoblotted (WB) against anti-ATPTb2 and anti-V5 peptide. Arrows indicate discernible bands corresponding to ∼40 kDa and ∼70 kDa. (C) Summary of oxidative phosphorylation and MICOS complex subunits that coimmunoprecipitate with Mic10-1–V5 identified by mass spectroscopy. Proteins in dark blue belong to the ATP synthase complex, those in orange belong to the MICOS complex, and those in yellow belong to the electron transport chain (ETC). The naming for the ATP synthase subunits was taken from Gahura et al. and Perez et al. (37, 47). The star indicates bait protein Mic10-1–V5. The intensity score is on the *x* axis (*n* = 3; error bars indicate SD). Pie charts on the right depict the portion of proteins identified versus the total number of subunits in each respective complex. Other proteins fitting the criteria are given in Fig. S2B in the supplemental material. (D) Sunburst chart of all proteins that coimmunoprecipitate with Mic10-1–V5 identified above the established threshold and criteria described in the text. Subunits of the ATP synthase complex are shown in dark blue and are further distinguished into the F_1_ and F_O_ moieties. Proteins belonging to the MICOS complex are shown in orange and are further separated into integral or peripheral membrane proteins. All other proteins that met the mass spectroscopy threshold are labeled by letters in boxes in the outside circle, which are defined in Fig. S2B. ETC complex cytochrome *c* oxidase (complex IV) is shown in yellow, and mitochondrial carriers are shown in green. Components of the tricarboxylic acid (TCA) cycle are in different shades of gray and are distinguished into SDH (succinate dehydrogenase), PDC (pyruvate dehydrogenase complex), and OGDC (oxoglutarate dehydrogenase complex), while those not belonging to any complex lack labels in the innermost circle. Other proteins that could not be categorized into a single group are shown in light blue.

To analyze whether any of the Mic10 paralogs interact with ATP synthase, the F_O_ moiety ATPTb2 was C-terminally V5 epitope tagged and used for coimmunoprecipitation (co-IP) from chemically cross-linked mitochondrial lysates. To facilitate the immunocapture of proteins interacting with the ATPTb2-V5 bait, hypotonically isolated mitochondria were cross-linked with dithiobis(succinimidyl propionate) (DSP) prior to solubilization. These were subsequently incubated with mouse anti-V5 antibody conjugated to protein G Dynabeads to immunoprecipitate the tag. After extensive washing, eluted proteins that coimmunoprecipitate with ATPTb2 were separated via protein electrophoresis and blotted, and the co-IP eluate was probed for the presence of Mic10-1 (the molecular weights of this and other proteins investigated here are given in Table S1). Mic10-1 was shown to coimmunoprecipitate with ATPTb2, yielding two discernible bands at approximately 35 kDa and 70 kDa (Fig. 2A). While un-cross-linked Mic10-1 is prominent in the input, it is completely absent in the eluate, suggesting that the protein can coimmunoprecipitate with ATPTb2 only when permanently linked to an unknown partner. We speculate that the ∼35-kDa band corresponds to an adduct between Mic10-1 and the unknown partner, and the ∼70-kDa band may represent the same adduct cross-linked to ATPTb2.

10.1128/mSphere.00327-21.5 TABLE S1 Encoding gene accession numbers and molecular weights of proteins mentioned in this study. Download Table S1, PDF file, 0.05 MB. Copyright © 2021 Cadena et al. 2021 Cadena et al. https://creativecommons.org/licenses/by/4.0/ This content is distributed under the terms of the Creative Commons Attribution 4.0 International license.

10.1128/mSphere.00327-21.2 FIG S2 Immunoprecipitation of the paralogs Mic10-1 and Mic10-2 (Fig. 2). (A) Immunoprecipitation (IP) with anti-V5 antibody of WT and Mic10-2–V5 mitochondria cross-linked with 80 μM dithiobis(succinimidyl propionate). The input (10% of the eluate) and eluates were resolved by SDS-PAGE and immunoblotted (WB) against anti-ATPTb2 and anti-V5. (B) Summary of the remaining proteins that coimmunoprecipitate in the Mic10-1–V5 pulldown identified by mass spectroscopy within the established threshold and criteria explained in the text. Mitochondrial protein carriers are in green, proteins that are components of the tricarboxylic acid (TCA) cycle are in gray, and proteins that cannot be categorized into a single group are in light blue (*n* = 3; error bars indicate SD). The intensity score is on the *y* axis. Download FIG S2, TIF file, 2.5 MB. Copyright © 2021 Cadena et al. 2021 Cadena et al. https://creativecommons.org/licenses/by/4.0/ This content is distributed under the terms of the Creative Commons Attribution 4.0 International license.

In order to verify the interaction between Mic10-1 and ATPTb2, we performed a reciprocal pulldown of C-terminally V5-epitope-tagged Mic10-1. Indeed, we were able to capture ATPTb2 with the Mic10-1–V5 bait in the eluted protein fraction with bands present at 40 kDa and 70 kDa. These bands were not detected in the eluate from Mic10-1–V5 immunoprecipitation (IP) after ATPTb2 was targeted by RNAi (Fig. 2B). The former band corresponds to un-cross-linked ATPTb2, and the latter most likely corresponds to the above-mentioned putative tripartite ATPTb2/Mic10-1/unknown partner adduct. Unlike Mic10-1, C-terminally V5-epitope-tagged Mic10-2 did not coimmunoprecipitate ATPTb2 (Fig. S2A), eliminating this paralog as an interaction partner with ATP synthase.

To investigate if Mic10-1 associates with the entire ATP synthase, rather than with its subcomplex or unassembled ATPTb2, proteins in the eluate from co-IP with Mic10-1–V5 were trypsinized and identified by liquid chromatography-tandem mass spectroscopy (LC-MS/MS). Mock IPs on cell lines lacking the V5 tag were performed in parallel as a negative control. Protein enrichment in comparison to mock IP controls was quantified using label-free quantification (LFQ) by a previously described pipeline (40). The identified proteins were filtered to meet the following criteria: (i) presence in all three triplicates, (ii) a mean of each triplicate’s log_2_-transformed LFQ intensity score of >23, (iii) absence from at least two out of three mock IPs, and (iv) presence within the ATOM40 depletome, which indicates that proteins are imported via this outer membrane translocator into the mitochondrion (41). In total, 51 proteins out of 209 detected proteins conformed to these criteria (Fig. 2C and D, Fig. S2B, and Data Set S1). Subunits of the ATP synthase complex and MICOS that are embedded or peripheral to the mitochondrial inner membrane (33) were the most represented proteins in the data set (Fig. 2D). Out of the 23 known subunits that make up the trypanosome ATP synthase, a total of 17 subunits were identified (Fig. 2C), including 5 of 6 subunits of F_1_. As expected, proteins belonging to the MICOS complex were also identified, with all integral subcomplex proteins being present alongside two components of the peripheral moiety; in total, six out of the nine MICOS proteins fit the criteria described above.

10.1128/mSphere.00327-21.7 DATA SET S1 List of proteins copurifying with Mic10-1 after chemical cross-linking (Fig. 2; see also Fig. S2 in the supplemental material). Shown is the Perseus software output Excel file of the label-free quantification (LFQ) of proteins enriched in Mic10-1–V5 immunoprecipitations (IPs) compared to a mock IP control. This file is appended with protein names (column A), presence in the ATOM40 depletome (column C), signifying being part of the mitoproteome (39), and whether a protein fits our criteria for bona fide enrichment as described in the text (column D). Results are filtered for “Yes” in column D but can be changed by the user by selecting the appropriate filter criteria accessed in each cell on the top row. Comparisons of LFQ scores between Mic10-1–V5 IP (v) and mock IP (c) are given in columns E to G. LFQ scores for Mic10-1–V5 and mock IPs for each detected protein are given in light blue columns H to N, whereas peptide counts are given in gray columns O to Y. The Mic10-1–V5 LFQ scores given in column K (highlighted in green cells with red bold font) were used for bar graphs in Fig. 2C and Fig. S2B and summarized in Fig. 2D. Download Data Set S1, XLSX file, 0.06 MB. Copyright © 2021 Cadena et al. 2021 Cadena et al. https://creativecommons.org/licenses/by/4.0/ This content is distributed under the terms of the Creative Commons Attribution 4.0 International license.

In contrast, only 1 protein from electron transport chain (ETC) complex IV (also known as cytochrome *c* oxidase) was recovered using the same criteria out of the 29 proteins that constitute the complex (42). Two other integral inner membrane proteins found to coimmunoprecipitate with the bait were mitochondrial carrier proteins, one of which was an ADP/ATP carrier protein (43). The other 25 proteins found within the set benchmark consisted mainly of highly abundant enzymes affiliated with the tricarboxylic acid (TCA) cycle as well as other prolific soluble mitochondrial matrix proteins (Fig. S2B). To conclude, we present evidence that Mic10-1 interacts with ATP synthase given that the complex’s subunits are enriched in Mic10-1–V5 IPs.

### Trypanosome ATPTb8 may be a functional analog of opisthokont F_1_F_O_-ATPase synthase dimer-enriched subunit e.

**FIG 3:**
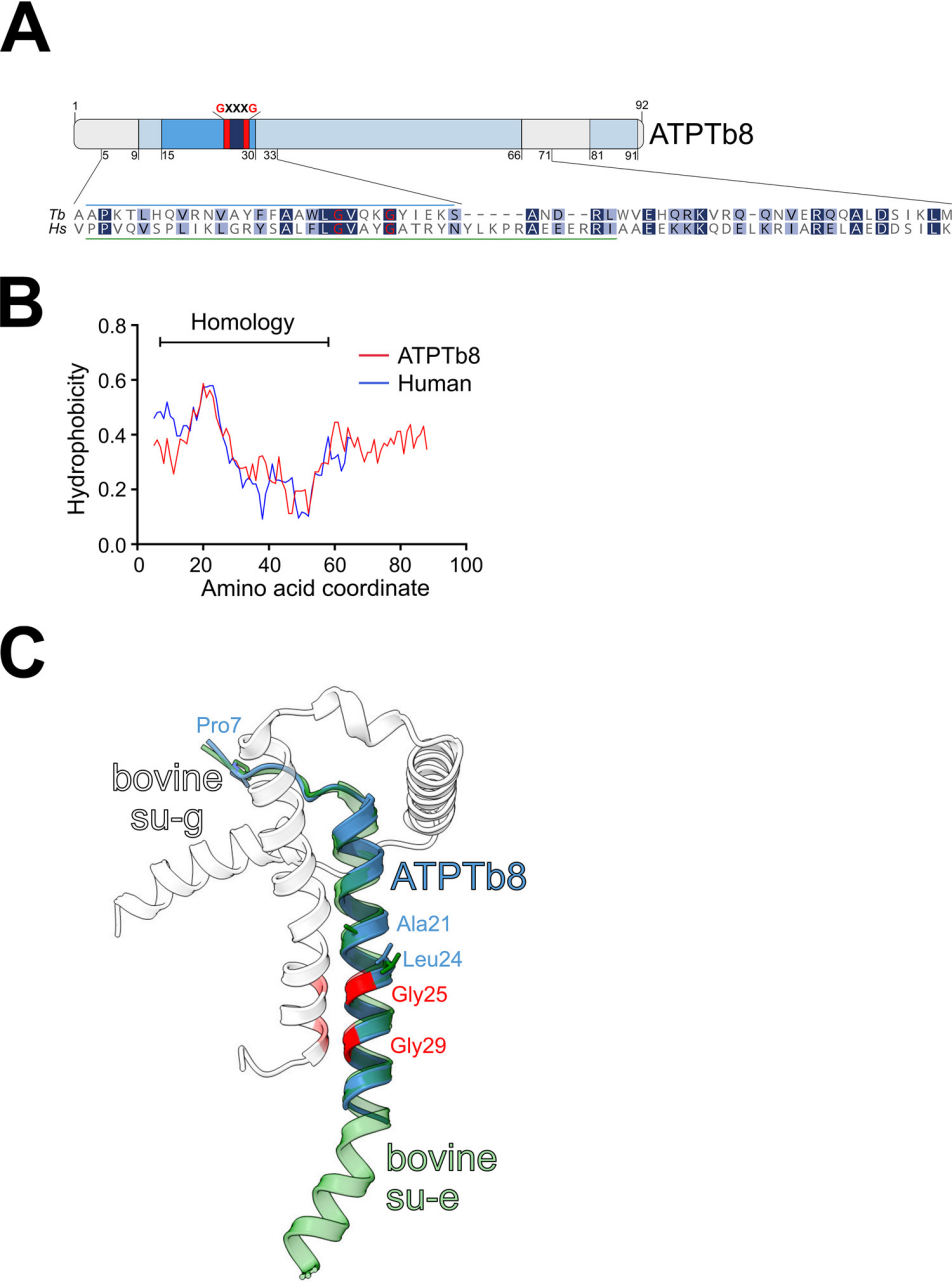
Trypanosome ATPTb8 may be a distant homolog or analog of opisthokont ATP synthase subunit e. (A) Schematic representation of critical features of the ATPTb8 primary structure. The predicted helical regions and the transmembrane domain are shown in pale and dark blue, respectively. The characteristic embedded GxxxG motif is labeled. A sequence alignment with human subunit e is shown for the region with similarity revealed by an HHpred search. The regions of ATPTb8 and mammalian subunit e depicted in panel C are marked with blue and green bars, respectively. (B) Kyte-Doolittle hydropathy profile comparison of ATPTb8 and the human homolog of subunit e. The range of homology between both sequences is shown as a bar at the top based on HHpred analysis. (C) Model of ATPTb8 predicted by SWISS-MODEL using the structure of bovine subunit e (su-e) as a template (PDB accession number 6ZPO) (44). The model is superposed with the bovine subunit e and subunit g (su-g) dimer. Glycines of all GxxxG motifs are in red. The conserved residues, which are involved in hydrophobic interactions between subunits e and g, are shown as sticks (44).

Previous studies in budding yeast demonstrated that Mic10 directly interacts with ATP synthase dimers via subunit e (28, 29), which is essential for the stability of dimers (20, 44, 45) but does not contribute directly to the monomer-monomer interface (46). In opisthokonts, subunit e contains a conserved GxxxG motif located within its single transmembrane domain (TMD) (45). Analyzing all the ATP synthase subunits identified in the Mic10-1 pulldown (Fig. 2C), we identified a low-molecular-weight protein, termed ATPTb8 (47), that contains this motif (Fig. 3A) and is highly conserved among all kinetoplastids (Fig. S3). A search with HHpred (48) revealed a similarity of the region of ATPTb8 encompassing the TMD to subunit e from yeast and human (also known as ATP5ME) (Fig. 3A). Additionally, a Kyte-Doolittle hydropathy plot comparison of ATPTb8 and human subunit e shows highly similar hydrophobicity profiles (Fig. 3B). SWISS-MODEL (49) identified mammalian subunit e among the best templates for structure homology modeling. The modeled part of ATPTb8 corresponds to the transmembrane region of mammalian subunit e, including the GxxxG motif, which homotypically interacts with its counterpart on subunit g (Fig. 3C). Just like subunit e, subunit g was implied in dimer stabilization in yeast (50), but recently, the protein was also proposed to make interdimer contacts in the rows of mammalian ATP synthases (51, 52).

10.1128/mSphere.00327-21.3 FIG S3 ATPTb8 is conserved among kinetoplastids (Fig. 3). The multiple-sequence alignment of ATPTb8 homologs from the order Kinetoplastida was performed with ClustalW using sequences obtained from TritrypDB.org. The GxxxG motif region is indicated by top brackets. The analysis of multiple-sequence alignment (AMSA) conservation score and consensus sequence are given on the bottom. The following species (TriTrypDB [https://tritrypdb.org] accession number; name) were used to generate the alignment: Crithidia fasciculata (CFAC1_220013400; ATPCf8), Leptomonas pyrrhocoris (LpyrH10_10_0680; ATPLp8), Leptomonas seymouri (Lsey_0093_0090; ATPLs8), Endotrypanum monterogeii (EMOLV88_250011100; ATPEm8), Leishmania braziliensis (LBRM2903_250012800; ATPLbr8), Leishmania panamensis (LPAL13_250010800; ATPLpa8), Leishmania enriettii (LENLEM3045_250011100; ATPLen8), Leishmania amazonensis (LAMA_000484200; ATPLam8), Leishmania mexicana (LmxM.25.0590; ATPLmx8), Leishmania infantum (LINF_250011100; ATPLin8), Leishmania donovani strain LV9 (LdBPK.25.2.000600; ATPLd8), Leishmania aethiopica (LAEL147_000405700; ATPLae8), Leishmania major (LMJLV39_250011600; ATPLm8), Leishmania tropica (LTRL590_250011700; ATPLt8), L. arabica (LARLEM1108_250011300; ATPLa8), Leishmania turanica (LTULEM423_250011500; ATPLtu8), Leishmania gerbilli (LGELEM452_250011200; ATPLge8), Blechomonas ayalai (Baya_001_1510; ATPBay8), Trypanosoma brucei TREU927 (Tb927.11.600; ATPTb8), Trypanosoma congolense (TcIL3000.A.H_000869900; ATPTco8), Trypanosoma vivax (TvY486_1100440; ATPTv8), Trypanosoma rangeli (TRSC58_00292; ATPTra8), Trypanosoma cruzi (C3747_9g1306c; ATPTc8), Paratrypanosoma confusum (PCON_0063970; ATPPcon8), and Bodo saltans (BSAL_59565; ATPBs8). Download FIG S3, TIF file, 2.0 MB. Copyright © 2021 Cadena et al. 2021 Cadena et al. https://creativecommons.org/licenses/by/4.0/ This content is distributed under the terms of the Creative Commons Attribution 4.0 International license.

**FIG 4:**
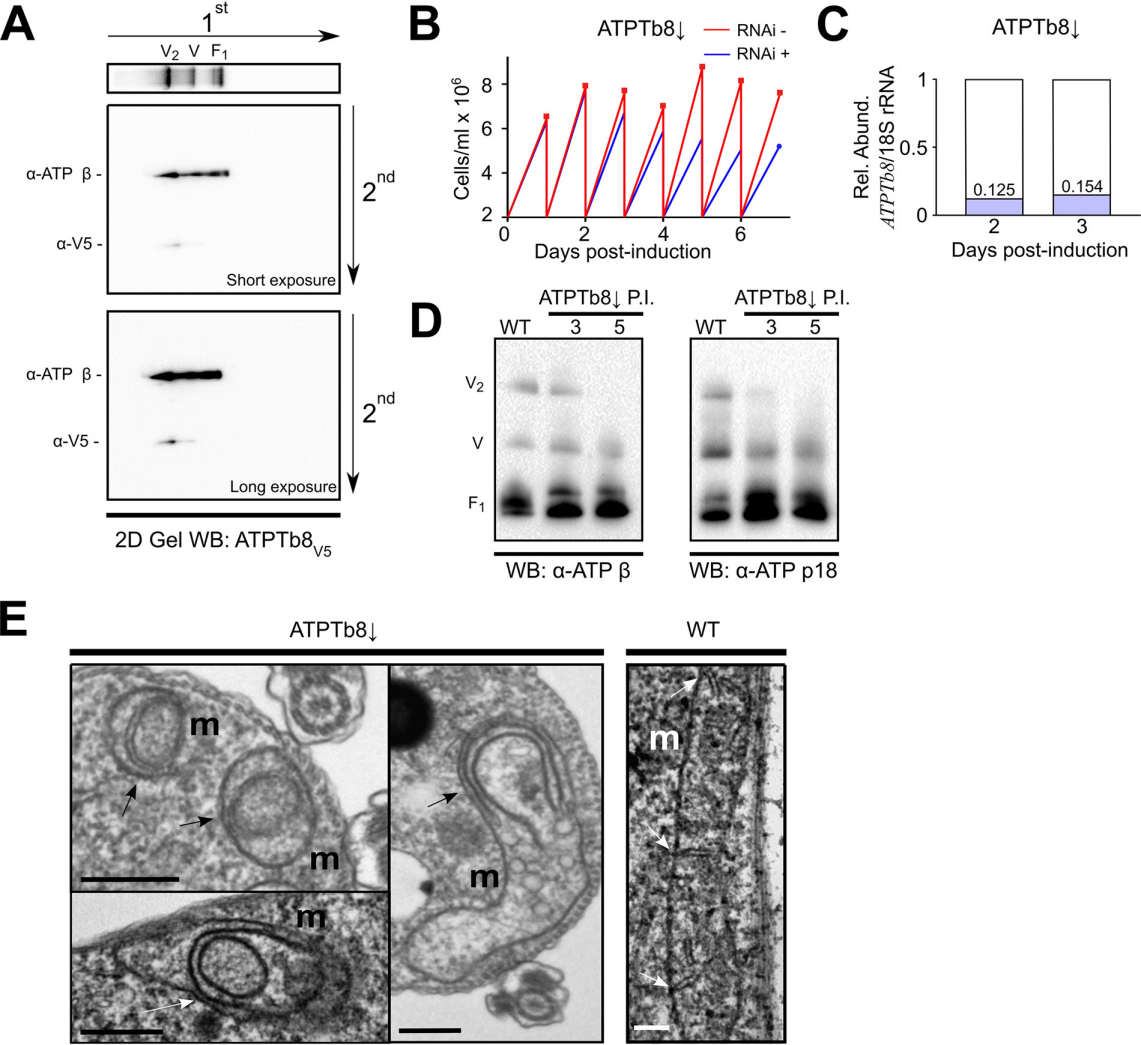
ATPTb8 is enriched in F_O_F_1_-ATP synthase dimers and crucial for crista formation. (A) Two-dimensional (2D) protein gel electrophoresis of ATPTb8-V5, with denaturing SDS-PAGE following first-dimensional BN-PAGE. (Top) First-dimension immunoblot (WB) against anti-ATP β showing the positions of ATP synthase dimers (V_2_), monomers (V), and the free F_1_ moiety. (Middle and bottom) Second-dimension immunoblots against anti-ATP β and anti-V5 at both short (middle) and long (bottom) exposures. (B) Measurement of ATPTb8↓ and negative-control cell growth in glucose-rich medium in which cells were diluted to 2 × 10^6^ cells/ml every day (*n* = 3; error bars indicate SD). The cell density is on the *y* axis; days postinduction are on the *x* axis. (C) Quantitative PCR verification of ATPTb8↓. The relative abundances of *ATPTb8* mRNA in RNAi-induced cell lines in comparison to uninduced control cells were normalized to the unaffected 18S rRNA at 2 and 3 days postinduction. (D) BN-PAGE of 1.5% *n-*dodecyl-β-d-maltoside-solubilized mitochondria from ATPTb8↓ cells at 3 and 5 days postinduction (P.I.) compared to the wild type (WT). Immunoblots (WB) against F_1_ subunit anti-ATP β and anti-ATP p18 are shown. (E) Transmission electron micrographs of WT T. brucei and ATPTb8↓ at 3 days postinduction. Arrows point to cristae of mitochondria (m). Bars, 500 nm.

Because the bioinformatic predictions indicated that ATPTb8 might be a distant homolog of opisthokont subunit e, we examined whether this subunit is required for the formation or stability of ATP synthase dimers in T. brucei. Utilizing two-dimensional (2D) protein gel electrophoresis, with denaturing SDS-PAGE following the first-dimensional BN-PAGE gel after treatment with 1.5% DDM, we show that C-terminally V5-tagged ATPTb8 is indeed predominantly detected in the dimer fraction (Fig. 4A), which is consistent with the hypothesis that ATPTb8 is involved in dimerization like yeast subunit e (24). Inducible RNAi silencing of ATPTb8 (ATPTb8↓) resulted in cellular growth arrest after 72 h (Fig. 4B and C). The growth defect in ATPTb8↓ is likely a consequence of compromised ATP production by oxidative phosphorylation, and it is consistent with growth phenotypes previously observed after silencing of other trypanosomal ATP synthase subunits (38, 39). No alterations in steady-state levels of Mic60 and Mic10-1 were detected in ATPTb8↓ inductions (Fig. S4). Noteworthy, the depletion of ATPTb8 preferentially affected the stability and/or assembly of ATP synthase dimers, as shown by Western blotting (WB) of BN-PAGE-resolved mitochondrial lysates probed with antibodies against subunits β and p18 (Fig. 4D).

10.1128/mSphere.00327-21.4 FIG S4 Characterization of the ATPTb8 RNAi cell line (Fig. 4). Shown is an immunoblot of ATPTb8↓ cells over the time course of RNAi, with antibodies indicated on the right. Days postinduction (P.I.) are shown at the top. Download FIG S4, TIF file, 0.2 MB. Copyright © 2021 Cadena et al. 2021 Cadena et al. https://creativecommons.org/licenses/by/4.0/ This content is distributed under the terms of the Creative Commons Attribution 4.0 International license.

To investigate whether the loss of ATPTb8 has an effect on mitochondrial morphology, transmission electron microscopy of cell sections was performed on cells after 3 days of RNAi induction. Mitochondria exhibiting a loss of this subunit depicted anomalous cristae that had circular or semicircular shapes (Fig. 4E), reminiscent of the onion-like structures that appear upon subunit e deletion in budding yeast (20, 26). Collectively, these data suggest that ATPTb8 is a dimer-incorporated subunit much in the same vein as subunit e in budding yeast.

### Trypanosome Mic10-1 interacts with dimeric F_1_F_O_-ATP synthase enriched with ATPTb8.

**FIG 5:**
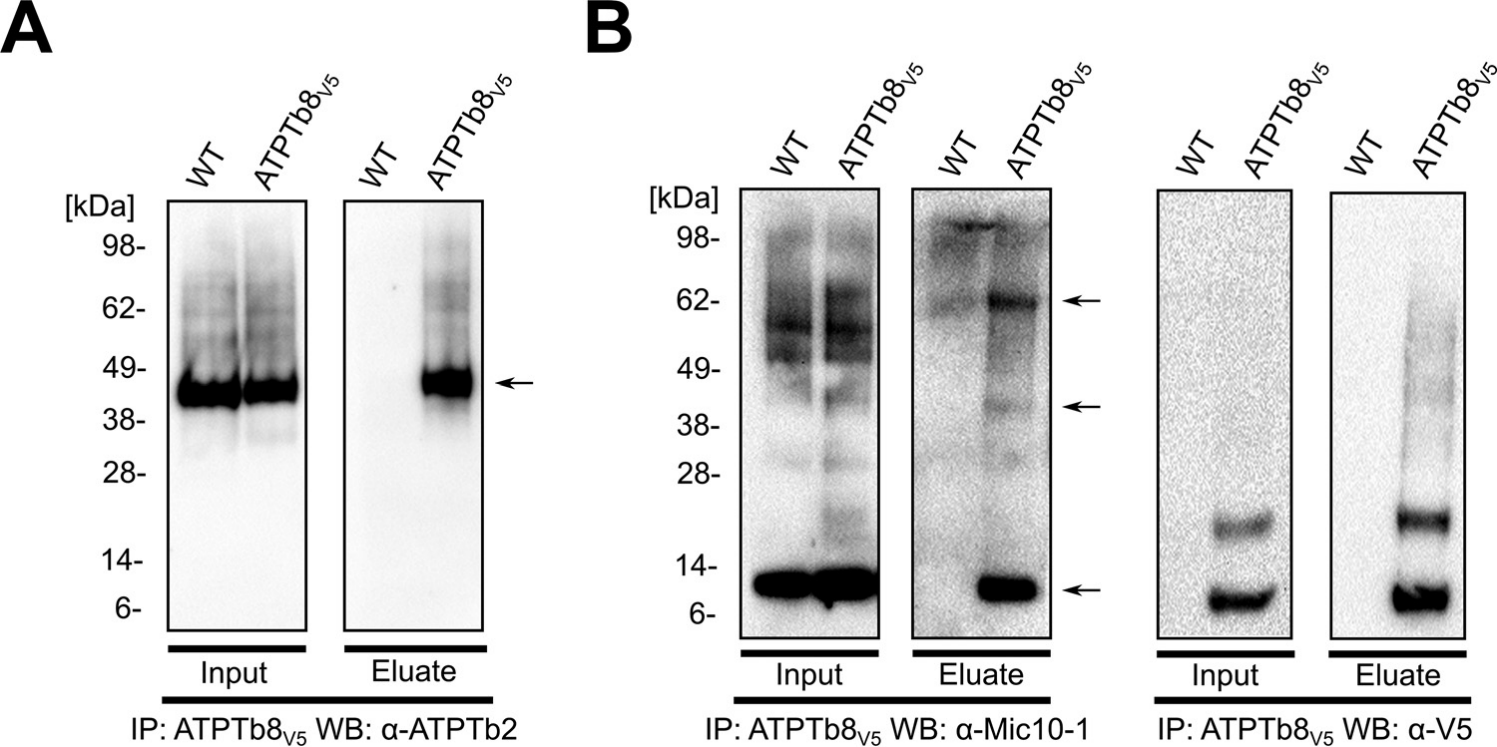
Mic10-1 interacts with F_O_F_1_-ATP synthase dimers. Immunoprecipitation (IP) of WT and ATPTb8-V5 mitochondria cross-linked as described in the legend of Fig. 2 was performed. (A) The input (10% of the eluate) and eluates were resolved by SDS-PAGE and immunoblotted (WB) using anti-ATPTb2 antibody. The arrow indicates a discernible band corresponding to ∼45 kDa (ATPTb2). (B) Same as panel A except using antibody against Mic10-1 and V5 peptide. Arrows indicate discernible bands corresponding to ∼10 kDa (Mic10-1), ∼40 kDa, and ∼70 kDa.

To confirm that Mic10-1 interacts with fully assembled ATP synthase dimers in T. brucei, ATPTb8 was *in situ* C-terminally V5 epitope tagged to perform a cross-link IP. Because ATPTb2 coimmunoprecipitated with ATPTb8-V5, the tag most likely does not interfere with the incorporation of the protein into ATP synthase (Fig. 5A). Next, we probed the IP eluate for the presence of Mic10-1, which was observed mostly as un-cross-linked (Fig. 5B), suggesting that Mic10-1 does not need to be tethered to any interaction partner to coimmunoprecipitate with ATPTb8, unlike with ATPTb2 (Fig. 2A). This further alludes to the possibility that Mic10-1 interacts more firmly with ATP synthase dimers enriched with ATPTb8. Interestingly, a faint band at 40 kDa and a stronger band at 70 kDa were also detected (Fig. 5B, left), reminiscent of the Mic10-1 immunoband pattern seen in the ATPTb2 IP (Fig. 2A).

## DISCUSSION

Crosstalk between MICOS and ATP synthase was first demonstrated in the budding yeast Saccharomyces cerevisiae, a model organism representing the supergroup Opisthokonta, which also encompasses humans. Deletion of Mic60 leads to an accumulation of nonionic-detergent-resistant ATP synthase oligomers, likely connected to the apparent functional antagonism between Mic60 and ATP synthase dimerization subunits e and g in yeast (30). At least a fraction of Mic10 interacts with subunit e, which appears to promote ATP synthase dimer oligomerization (28, 29). Indeed, these two phenomena were proposed to be linked in the Mic60 deletion mutants: the cumulative effect of MICOS disruption with the remaining Mic10’s stabilizing activity on ATP synthase dimer oligomers resulting in the observed hyperoligomerization phenotype (3, 28).

The physiological role of MICOS-ATP synthase crosstalk in yeast mitochondria remains enigmatic (3), precluding any rational means to hypothesize whether this can be a general eukaryotic phenomenon and not just a fungal novelty. In this work, we have documented the interplay between MICOS and ATP synthase dimers in T. brucei, an experimental model belonging to the clade Discoba, which separated from the Opisthokonta ∼1.8 billion years ago (23, 31). Even though particular aspects of these complexes are conserved between yeast and trypanosomes, such as MICOS maintaining crista junctions (32) and rows of ATP synthase dimers occurring at the rims of discoidal cristae (22), both complexes exhibit numerous divergent features (30, 37). Based on the early branching of the two lineages and the marked divergence of their MICOS and ATP synthase, we reason that the crosstalk between MICOS and ATP synthase is a fundamental and ancestral property of cristae.

Specifically, we have demonstrated that Mic60 depletion in T. brucei phenocopies the stabilization of ATP synthase oligomerization observed in S. cerevisiae Mic60 deletion mutants. In addition to being our first indication that there is MICOS-ATP synthase crosstalk in T. brucei, this result also supports the putative designation of this subunit as Mic60 despite its lack of a mitofilin domain (32).

We show that crosstalk between MICOS and ATP synthase is mediated by one of the two trypanosome Mic10 paralogs, Mic10-1. We demonstrate that Mic10-1 cross-links to ATPTb2 and ATPTb8, two membrane-associated subunits of the F_O_ moiety (34, 37). This observation is consistent with Mic10-1 being an integral membrane protein mostly comprised of two TMDs (32). Furthermore, Mic10-1 coimmunoprecipitates most of the ATP synthase subunits after cross-linking. However, ATP synthase subunits were scarcely detected when MICOS subunits were immunoprecipitated in the absence of any cross-linker (32), suggesting this intercomplex interaction is dynamic and perhaps transient (53).

Our results demonstrate that there are functional differences between Mic10-1 and Mic10-2, as the latter does not interact with ATP synthase. Because Mic10-1 mediates this crosstalk as Mic10 does in yeast, it may represent the conventional Mic10 paralog, while Mic10-2 may be the diversified variant whose precise role in discoidal crista shaping remains undefined. Indeed, trypanosome Mic10-1 has the typical GxGxGxG glycine-rich motif in the C-terminal TMD, a property shared with other Mic10 homologs, whereas Mic10-2’s corresponding TMD has a reduced GxGxG motif (32). It is tempting to speculate that this difference may be responsible for Mic10-1 interacting with ATP synthase and may explain why Mic10-2 does not. However, other factors instead of or in synergy with the GxGxGxG motif could possibly mediate Mic10’s interaction with ATP synthase.

Because yeast Mic10 interacts with ATP synthase dimers, we set out to identify any subunits that may potentially affect dimerization and thus serve as a marker for this higher-order configuration. The F_O_ moiety subunits of T. brucei ATP synthase identified by mass spectroscopy have not been fully characterized to date (37). Thus, we screened these subunits for the presence of a single-pass TMD with a GxxxG motif, a signature of yeast subunit e (24, 45). The protein ATPTb8 emerged from this screen, and outputs of structural modeling and hidden Markov searches support potential homology with human subunit e. ATPTb8 was enriched in dimer fractions, and its ablation phenotype is highly evocative of that of yeast subunit e, in which ATP synthase dimers become sensitive to nonionic detergent treatment along with the correlative emergence of defective cristae (25). Thus, we predict that ATPTb8 may represent a homolog of subunit e. Whether discoban ATPTb8 and opisthokont subunit e arose through divergent or convergent evolution remains an open question. Phylogenetic analysis may be precluded by the short lengths of these polypeptides and a high degree of divergence, as observed in human and yeast homologs of subunit e, despite belonging to the same eukaryotic supergroup.

**FIG 6:**
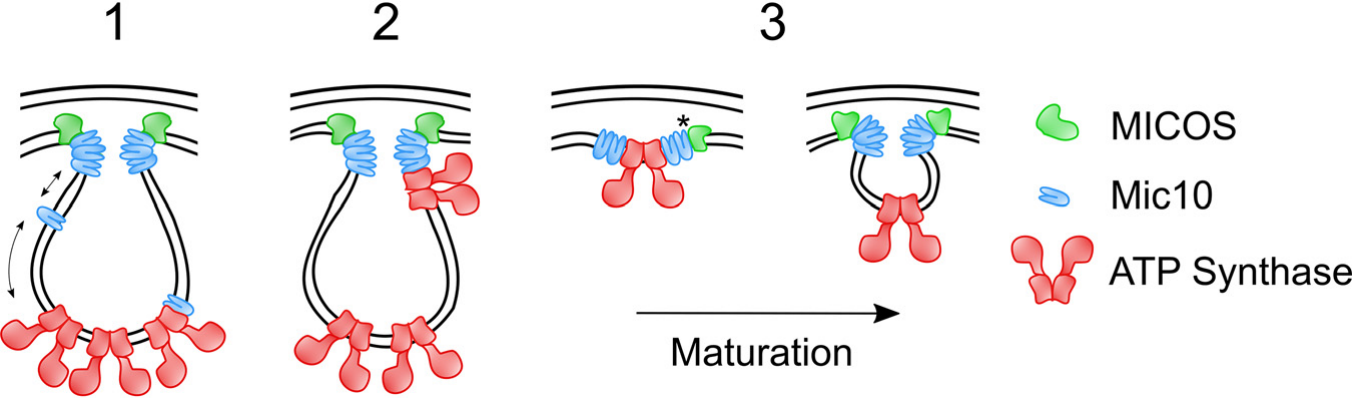
Possible modes for the functional interplay between MICOS and F_O_F_1_-ATP synthase dimers in eukaryotes. Shown is a schematic representation of 3 possible mechanisms of the functional interplay between MICOS (green) and F_O_F_1_-ATP synthase (red) in the mitochondrial inner membrane. (1) An extra-MICOS fraction of Mic10 (blue) interacts with ATP synthase dimers at crista rims. Arrows represent crosstalk between MICOS and ATP synthase dimers via Mic10. (2) Mic10 bridges the MICOS complex to ATP synthase dimers directly at crista junctions, enabling dimers to induce positive curvature at the tubular necks of cristae. (3) Transient Mic10-1 interacts with ATP synthase at nascent cristae during ATP synthase dimer-induced invaginations. The asterisk represents a putative MICOS complex subassembly. See Discussion for a more in-depth description of each scenario.

Why is this crosstalk between MICOS and F_1_F_O_-ATP synthase present in both opisthokonts and discobans, and what role does it play? Two different hypotheses have been previously given, and here, we propose a third (Fig. 6). These hypotheses may not necessarily be mutually exclusive and perhaps may not apply to all lineages. The first hypothesis postulates that an extra-MICOS fraction of Mic10 interacts with ATP synthase dimers. The local negative curvature induced by Mic10 (15, 16) may relieve membrane tension caused by the positive bending mediated by dimer rows (54), stabilizing the ATP synthase oligomers. In addition, Mic10 binding might enable coordination between MICOS and ATP synthase to temporarily and spatially regulate the shape of cristae (3, 28). In the second hypothesis, Mic10 acts to bridge the MICOS complex with ATP synthase dimers directly at crista junctions (29). In this scenario, the ATP synthase dimers would induce positive curvature of tubular necks of cristae. However, it should be noted that crista junctions in S. cerevisiae and other fungi have a predominantly slot-like morphology mainly comprised of flat membranes (9). Our third hypothesis is that we are capturing a transient Mic10-1 interaction with ATP synthase occurring at nascent cristae during the process of ATP synthase dimer-induced invagination of the inner membrane at the future site of crista junctions. This interaction may involve Mic10 alone or as part of a putative MICOS subassembly present on emerging cristae. Why the occurrence of this crosstalk has been preserved throughout the diversification of eukaryotes is still waiting to be answered.

## MATERIALS AND METHODS

### Generation of transgenic cell lines.

All trypanosome cell lines used in this study were derived from T. brucei SmOxP927 procyclic-form cells, i.e., TREU 927/4 expressing T7 RNA polymerase and a tetracycline repressor to allow inducible expression (55), and are listed in Table S2 in the supplemental material. The C-terminally tagged ATPTb8-3×V5 and ATPTb2-3×V5 cell lines were generated using the pPOTv4 vector containing a hygromycin resistance cassette by PCR amplification with the oligonucleotides based on a previously established protocol (56) (Table S2). The underlined sequence anneals to the pPOT vector.

10.1128/mSphere.00327-21.6 TABLE S2 Key resource table. Download Table S2, PDF file, 0.1 MB. Copyright © 2021 Cadena et al. 2021 Cadena et al. https://creativecommons.org/licenses/by/4.0/ This content is distributed under the terms of the Creative Commons Attribution 4.0 International license.

ATPTb8 RNAi constructs were made by PCR amplifying a fragment of the ATPTb8 gene from T. brucei strain 927 genomic DNA with the oligonucleotides listed in Table S2. Utilizing the underlined XhoI and BamHI restriction sites within the primer, these fragments were cloned into the pAZ055 stem-loop RNAi vector (Table S2).

### Trypanosome cell culture.

Cells were grown at 27°C in SDM-79 medium supplemented with 10% (vol/vol) fetal bovine serum and 7.5 mg/liter hemin. Cells were grown in the presence and absence of 1 μg/ml doxycycline for RNAi induction. Cell density was measured using the Beckman Coulter Z2 cell and particle counter and maintained at the exponential mid-log growth phase throughout the analyses.

### SDS-PAGE and Western blotting.

Protein samples were separated on a Bolt 4 to 12% Bis-Tris Plus gel (Invitrogen), blotted onto a polyvinylidene difluoride (PVDF) membrane (Amersham), blocked in 5% milk, and probed with the appropriate primary antibody listed in Table S2 diluted in 5% milk in phosphate-buffered saline plus Tween (PBS-T). This was followed by incubation with a secondary horseradish peroxidase (HRP)-conjugated anti-rabbit or anti-mouse antibody (1:2,000; Bio-Rad), depending on the origin of the primary antibody. Proteins were visualized using the Pierce ECL system (Genetica/Bio-Rad) on a ChemiDoc instrument (Bio-Rad).

### Blue native PAGE, in-gel histochemical staining of F_1_-ATPase activity, and 2D electrophoresis.

Blue native PAGE (BN-PAGE) of mitochondrial lysates was adapted from previously reported protocols (34). Briefly, mitochondrial vesicles from ∼5 × 10^8^ cells were resuspended in 100 μl NativePAGE sample buffer (Invitrogen), lysed with 1.5% (vol/vol) dodecylmaltoside (DDM) for 20 min on ice, and then cleared by centrifugation (18,600 × *g* for 15 min at 4°C). The protein concentration of each lysate was determined by a Bradford assay (57) so that 50 μg of total protein could be mixed with 5% Coomassie brilliant blue G-250 before loading on a 3 to 12% Bis-Tris BNE gel (Invitrogen). After electrophoresis (2.5 h at 150 V at 4°C), the gel was either blotted onto a PVDF membrane (Amersham) or incubated overnight in an ATPase reaction buffer [35 mM Tris (pH 8.0), 270 mM glycine, 19 mM MgSO_4_, 0.5% Pb(NO_3_)_2_, 15 mM ATP]. For 2D electrophoresis, individual BNE lanes were cut and placed horizontally on a 12% polyacrylamide gel prior to electrophoresis and Western blot analysis.

### Hypotonic mitochondrial isolation.

Mitochondrial vesicles were obtained by hypotonic lysis as described previously (32). Briefly, cell pellets from 5 × 10^8^ cells were washed in SBG (150 mM NaCl, 20 mM glucose, 1.6 mM NaHPO_4_), resuspended in DTE (1 mM Tris, 1 mM EDTA [pH 8.0]), and disrupted through a 25-gauge needle. To reintroduce a physiologically isotonic environment, disrupted cells were immediately added to 60% sucrose. Samples were centrifuged (12,300 × *g* for 15 min at 4°C) to clear the soluble cytoplasmic material from pelleted mitochondrial vesicles. The resulting pellets were resuspended in STM (250 mM sucrose, 20 mM Tris [pH 8.0], 2 mM MgCl_2_) and incubated with 5 μg/ml DNase I for 1 h on ice. An equal volume of STE buffer (250 mM sucrose, 20 mM Tris [pH 8.0], 2 mM EDTA [pH 8.0]) was subsequently added, and the mixture was then centrifuged (18,600 × *g* for 15 min at 4°C). Pellets enriched with mitochondrial vesicles were then snap-frozen in liquid nitrogen for further analysis or subsequently treated with a chemical cross-linker, as described below.

### Chemical cross-linking.

Hypotonically isolated mitochondrial lysates were resuspended in 1 ml PBS (pH 7.5) and incubated with 80 μM DSP for 2 h on ice. The reaction was stopped by the addition of 20 mM Tris-HCl (pH 7.7) for 15 min at room temperature (RT), and the lysates were then cleared by centrifugation (18,600 × *g* for 15 min at 4°C). The cross-linked mitochondrial vesicles were then snap-frozen for subsequent immunoprecipitation (IP).

### Immunoprecipitations.

IP of tagged proteins was adapted from previously reported protocols (32). In brief, DSP-cross-linked mitochondrial vesicles from ∼5 × 10^8^ cells were solubilized in IPP50 (50 mM KCl, 20 mM Tris-HCl [pH 7.7], 3 mM MgCl_2_, 10% glycerol, 1 mM phenylmethanesulfonyl fluoride [PMSF], complete EDTA-free protease inhibitor cocktail [Roche]) supplemented with 1% (vol/vol) Igepal for 20 min on ice. After centrifugation (18,600 × *g* for 15 min at 4°C), the supernatant was added to 1.5 mg of anti-V5-conjugated magnetic beads, previously washed three times in 200 μl of IPP50 plus 1% Igepal for 5 min at RT. The solubilized mitochondria were rotated with beads for 90 min at 4°C. After the removal of the flowthrough, the beads were washed three times in IPP50 plus 1% Igepal. Prior to elution, the beads were transferred into a new tube. Elution was done in 0.1 M glycine (pH 2.0) for 10 min at 70°C with shaking at 1,000 rpm. The eluate was neutralized with 1 M Tris (pH 8.0). The elution step was repeated to achieve higher recovery. The eluates were further processed for LC-MS/MS analysis or resolved by SDS-PAGE. IPs were performed in triplicate.

### Protein preparation and mass spectroscopy.

Triplicate eluates of coimmunoprecipitated proteins were processed for mass spectroscopy analysis as described previously (58, 59). In brief, samples were resuspended in 100 mM tetraethylammonium bromide (TEAB) containing 2% sodium deoxycholate (SDC). Cysteines were reduced with a final concentration of 10 mM Tris(2-carboxyethyl)phosphine hydrochloride (TCEP) and subsequently cleaved with 1 μg trypsin overnight at 37°C. After digestion, 1% trifluoroacetic acid (TFA) was added to wash twice, and eluates were resuspended in 20 μl of TFA per 100 μg of protein. A nano-reversed-phased column (Easy-Spray column, 50-cm by 75-μm inner diameter, PepMap C_18_, 2-μm particles, 100-Å pore size) was used for LC-MS analysis. Mobile phase buffer A consisted of water and 0.1% formic acid. Mobile phase B consisted of acetonitrile and 0.1% formic acid. Samples were loaded onto the trap column (Acclaim PepMap300 C_18_, 5 μm, 300-Å-wide pore, 300 μm by 5 mm) at a flow rate of 15 μl/min. The loading buffer consisted of water, 2% acetonitrile, and 0.1% TFA. Peptides were eluted using a mobile phase B gradient from 2% to 40% over 60 min at a flow rate of 300 nl/min. The peptide cations eluted were converted to gas-phase ions via electrospray ionization and analyzed on a Thermo Orbitrap Fusion instrument (Q-OT-qIT; Thermo Fisher). Full MS spectra were acquired in the Orbitrap instrument with a mass range of 350 to 1,400 *m/z*, at a resolution of 120,000 at 200 *m/z*, and with a maximum injection time of 50 ms. Tandem MS was performed by isolation at 1.5 Th with the quadrupole, high-energy collisional dissociation (HCD) fragmentation with a normalized collision energy of 30, and rapid-scan MS analysis in the ion trap. The MS/MS ion count target was set to 10^4^, and the maximum infection time was set at 35 ms. Only those precursors with a charge state of 2 to 6 were sampled. The dynamic exclusion duration was set to 45 s with a 10-ppm tolerance around the selected precursor and its isotopes. Monoisotopic precursor selection was on with a top-speed mode of 2-s cycles.

### Analysis of mass spectrometry peptides.

Label-free quantification of the data was performed using MaxQuant software (version 1.6.2.1) (60). The false discovery rates for peptides and proteins were set to 1% with a specified minimum peptide length of 7 amino acids. The Andromeda search engine was used for the MS/MS spectra against the Trypanosoma brucei database (downloaded from UniProt, November 2018, containing 8,306 entries). Enzyme specificity was set to C-terminal Arg and Lys, alongside cleavage at proline bonds, with a maximum of 2 missed cleavages. Dithiomethylation of cysteine was selected as a fixed modification, with N-terminal protein acetylation and methionine oxidation as variable modifications. The “match between runs” feature in MaxQuant was used to transfer identification to other LC-MS/MS runs based on mass and retention time with a maximum deviation of 0.7 min. Quantifications were performed using a label-free algorithm as described recently (60). Data analysis was performed using Perseus software (version 1.6.1.3). Only proteins identified exclusively alongside a mean log_2_-transformed LFQ intensity score of >23 and found in the ATOM40 depletome (indicating that proteins are imported into the mitochondria) were considered putative interaction proteins (41). Exclusive identification is defined here as a situation where a given protein was measured in all three replicates of the bait protein pulldown but absent in at least two out of three control replicates.

### Transmission electron microscopy.

For ultrastructural studies, cells were centrifuged at 620 × *g* for 10 min at RT and immediately fixed with 2.5% glutaraldehyde in 0.1 M phosphate buffer (pH 7.2). Samples were then postfixed in osmium tetroxide for 2 h at 4°C, washed, dehydrated through an acetone series, and embedded in resin (Polybed 812; Polysciences, Inc.). A series of ultrathin sections were cut using a Leica UCT ultramicrotome (Leica Microsystems) and counterstained with uranyl acetate and lead citrate. Samples were observed using a JEOL 1010 transmission electron microscope operating at an accelerating voltage of 80 kV and equipped with a MegaView III charge-coupled-device (CCD) camera (Emsis).

### Bioinformatic analysis.

The multiple-sequence alignment of ATPTb8 homologs from the order Kinetoplastida was performed by ClustalW using default settings. These alignments were trimmed to remove gaps and regions of poor conservation and rendered in Jalview (version 2.11.1.3) (61). Sequences were obtained from the TriTrypDB database. The regions homologous to human and yeast subunit e were determined using the HHpred toolkit (48), and the helical region and transmembrane domain within the ATPTb8 sequence were predicted by PSIPRED 4.0 and MEMSAT-SVM software (62), respectively. Kyte-Doolittle hydropathy plots for ATPTb8 and the human subunit e sequence were calculated using the ProtScale prediction software courtesy of the ExPASy server (63). The structure of ATPTb8 was homology modeled using SWISS-MODEL (49).

### Data availability.

The mass spectroscopy data have been deposited to the ProteomeXchange Consortium (http://www.proteomexchange.org) via the PRIDE partner repository with the data set identifier PXD025109.
