# Erratum for Cadena et al., “Mitochondrial Contact Site and Cristae Organization System and F_1_F_O_-ATP Synthase Crosstalk Is a Fundamental Property of Mitochondrial Cristae”

**DOI:** 10.1128/msphere.00189-22

**Published:** 2022-05-02

**Authors:** Lawrence Rudy Cadena, Ondřej Gahura, Brian Panicucci, Alena Zíková, Hassan Hashimi

**Affiliations:** a Institute of Parasitology, Biology Center, Czech Academy of Sciences, České Budějovice, Czech Republic; b Faculty of Science, University of South Bohemia, České Budějovice, Czech Republic

## ERRATUM

Volume 6, no. 3, e00327-21, 2021, https://doi.org/10.1128/mSphere.00327-21. The second paragraph of Acknowledgments should read as follows: “We gratefully acknowledge funding from the following sources: Czech Science Foundation grants 20-23513S to H.H., 18-17529S to A.Z., and 20-04150Y to O.G.; Czech Ministry of Education grant OPVVV16_019/0000759; and Czech BioImaging grant LM2015062.”

