# Supplementary figures and images for: Mitochondrial Contact Site and Cristae Organization System and F1FO-ATP Synthase Crosstalk Is a Fundamental Property of Mitochondrial Cristae
Source: mSphere. 2021 Jun 16;6(3):e00327-21. doi: 10.1128/mSphere.00327-21 (PMC8265648; doi:10.1128/mSphere.00327-21)

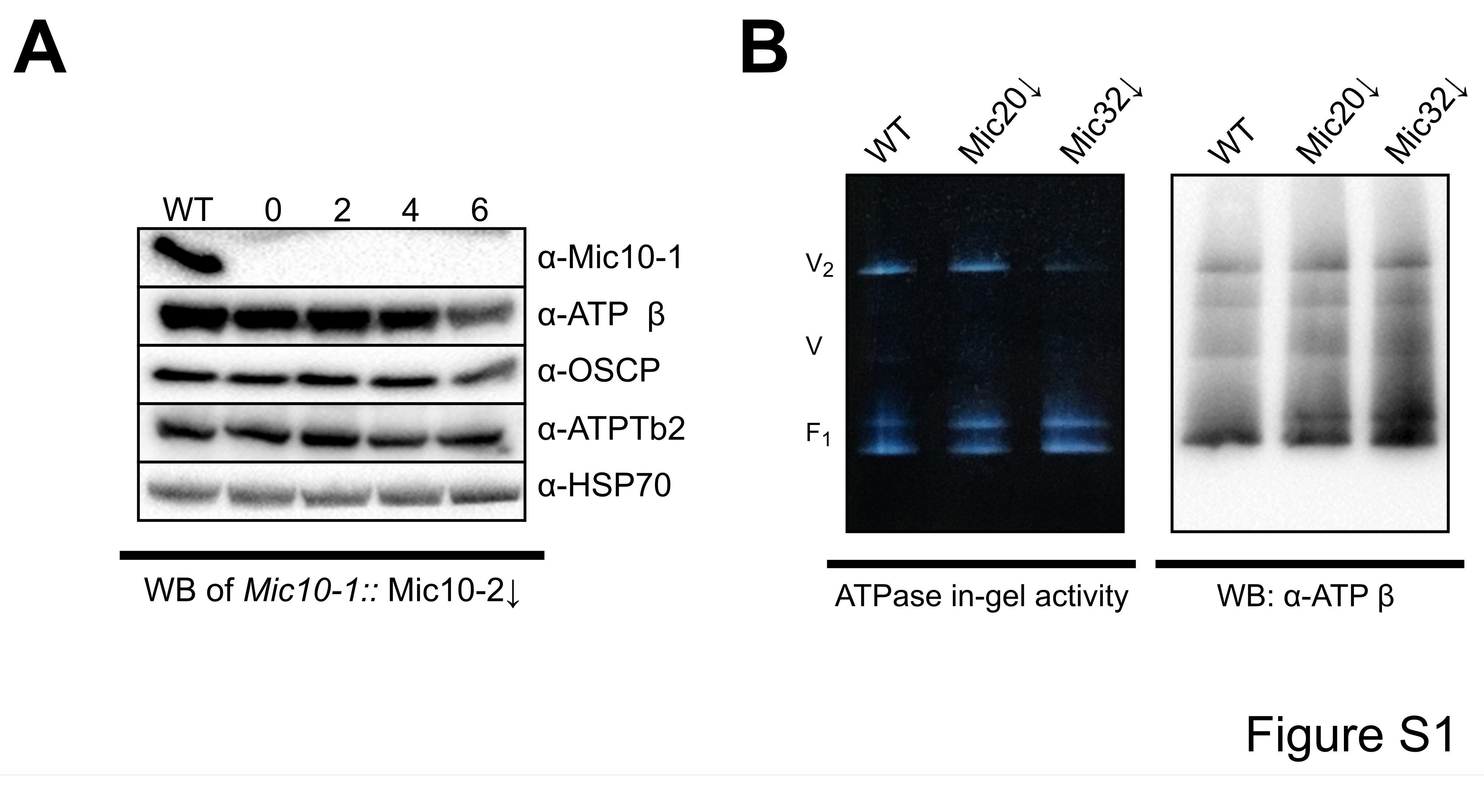

Supplement: FIG S1 [file msphere.00327-21-sf001.tif]

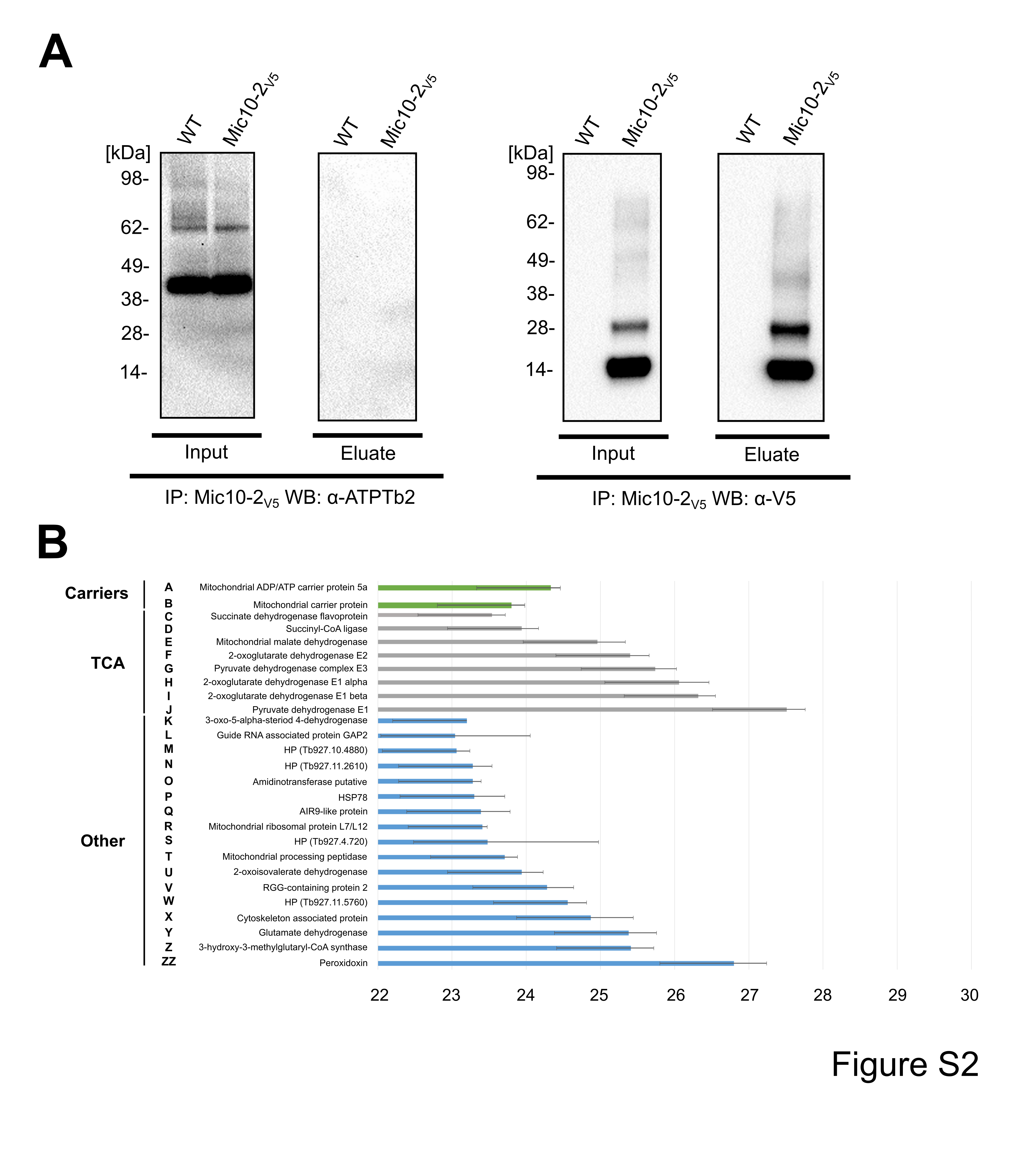

Supplement: FIG S2 [file msphere.00327-21-sf002.tif]

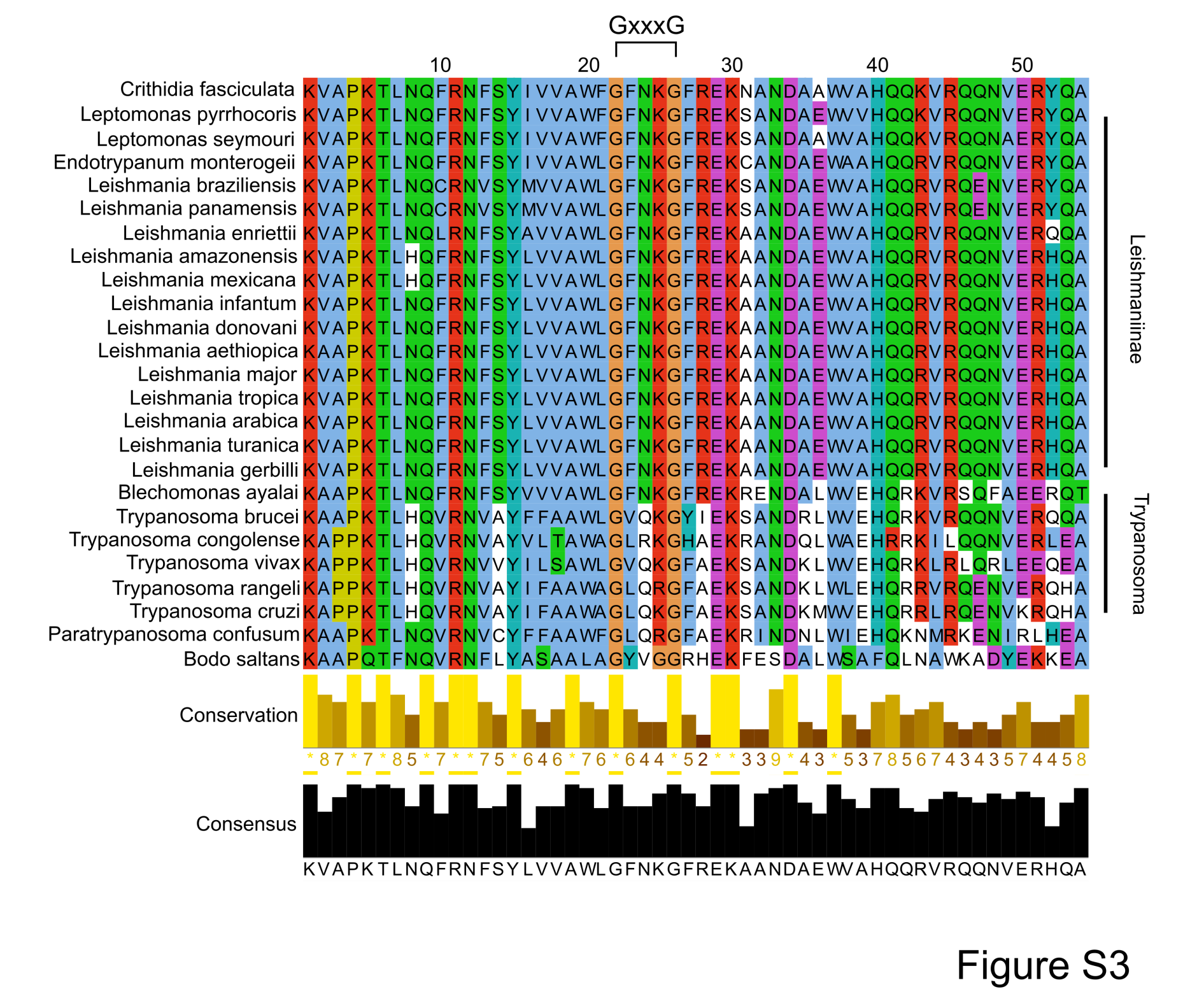

Supplement: FIG S3 [file msphere.00327-21-sf003.tif]

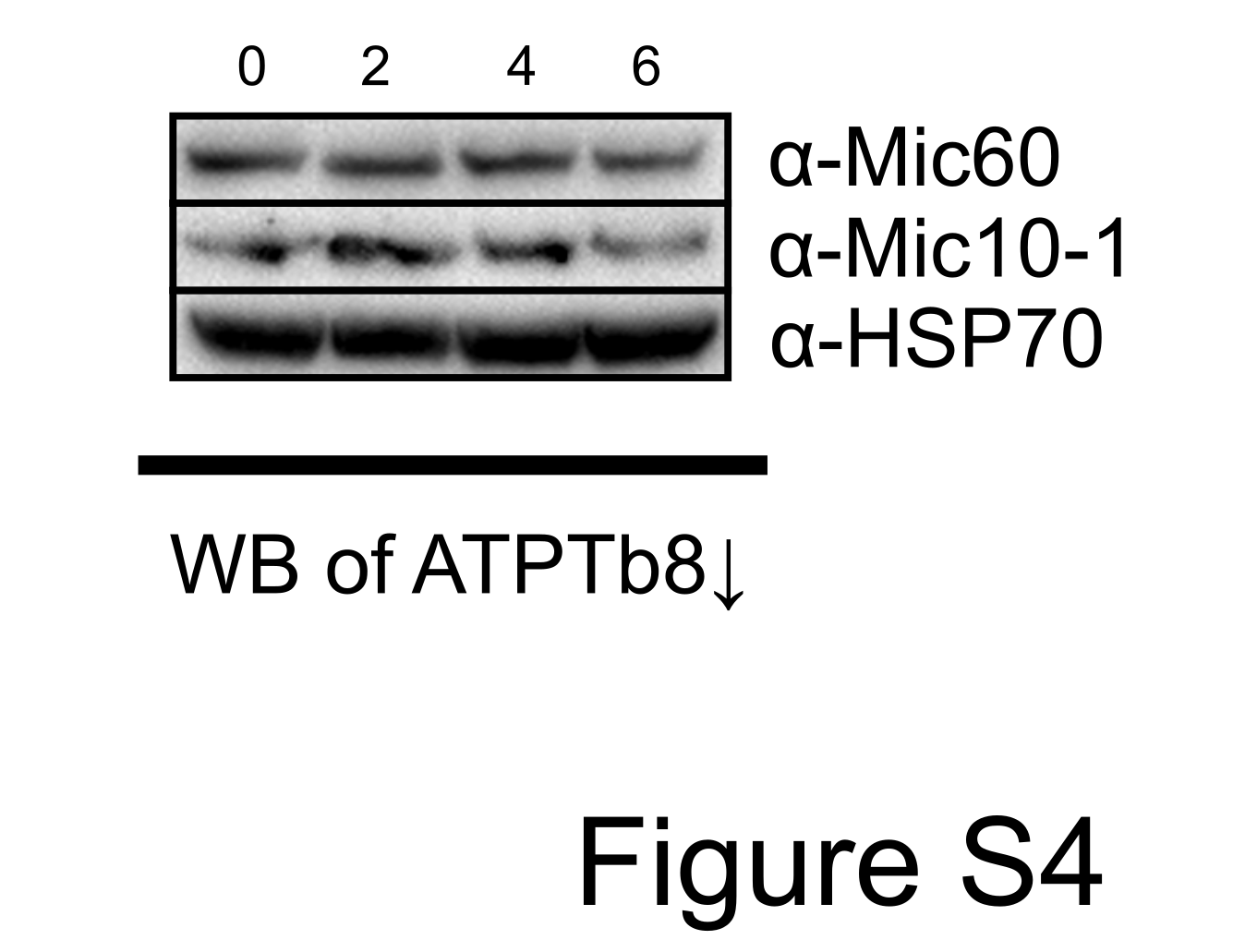

Supplement: FIG S4 [file msphere.00327-21-sf004.tif]
