# Supplementary material for: Mitochondrial Contact Site and Cristae Organization System and F1FO-ATP Synthase Crosstalk Is a Fundamental Property of Mitochondrial Cristae
Source: mSphere. 2021 Jun 16;6(3):e00327-21. doi: 10.1128/mSphere.00327-21 (PMC8265648; doi:10.1128/mSphere.00327-21)
Supplement: TABLE S1 [file msphere.00327-21-st001.pdf]

**Table S1:** Encoding gene accession numbers and molecular weights of proteins mentioned in this study.

| Name | Gene ID | Molecular weight [kDa] |
|---|---|---|
| Mic10-1 | Tb927.10.1580 | 12.2 |
| Mic10-2 | Tb927.11.2190 | 11.2 |
| Mic60 | Tb927.9.10160 | 25.4 |
| Mic32 | Tb927.2.2940 | 32.2 |
| Mic20 | Tb927.10.11900 | 19.5 |
| ATPTb8 | Tb927.11.600 | 10.4 |
| ATPTb2 | Tb927.5.2930 | 43.3 |
| ATP β | Tb927.3.1380 | 55.8 |
| ATPp18 | Tb927.5.1710 | 21.2 |
| OSCP | Tb927.6.4990 | 20.1 |
| mtHSP70 | Tb927.6.3740 | 71.5 |
