# Supplementary material for: Mitochondrial Contact Site and Cristae Organization System and F1FO-ATP Synthase Crosstalk Is a Fundamental Property of Mitochondrial Cristae
Source: mSphere. 2021 Jun 16;6(3):e00327-21. doi: 10.1128/mSphere.00327-21 (PMC8265648; doi:10.1128/mSphere.00327-21)
Supplement: TABLE S2 [file msphere.00327-21-st002.pdf]

Table S2. Key resources table.

T. brucei **experimental models (RRID: SCR_004786)**	
**Cell line**	**Reference (see main text reference list)**
Mic10-1-3xV5	(31)
Mic10-2-3xV5	(31)
ΔMic10-1:Mic10-2↓	(31)
Mic60↓	(31)
Mic32↓	(31)
Mic20↓	(31)
ATPTb2-3xV5	This study
ATPTb2↓	(K. Šubrtová, B. Panicucci, and A. Ziková, PLOS Pathog. 10(2): e1004660, 2015, https://doi10.1371/Journal.ppat.1004660)
ATPTb8-3xV5	This study
ATPTb8↓	This study
Mic10-1-3xV5-ATPTb2↓	This study
Mic10-1-3xV5-ATPTb8↓	This study

**Recombinant DNA**	
**Plasmid**	**Reference (see main text reference list)**
pPOT-V5-HygR (*in situ* epitope tagging)	(31)
pAZ055-PhleoR (stem loop RNAi vector)	This study

**Oligonucleotides (see main text for explanation of underlined sequences)**		
**Amplicon**	**Forward PCR primer (5'→3')**	**Reverse PCR primer (5'→3')**
ATPTb8-3xV5 epitope tagging	AAGGCGTCAGGGCCACCACTCCAGGTCAGCTTGAAGGTGTGCCCGCAGAGCTCCAGCAGCTTGCTGAAGCTTTCACTAAGGGTTCTGGTAGTGGTTCC	CGCCCACCCAAGTGTGTCCCTTTTCCACCATCCCGGACCGAAAGGAGGGGAGAAGGGGAGGGGTGCCCAATTGTTTCCTACCAATTTGAGAGACCTGTGC
ATPTb2-3xV5 epitope tagging	TTCTTGCCCTCCAGGCTCGCATTAATCAGCGATGAGGTTGAGCCTTCACAAACCGAACAAAAGAAAAAGGCCCATCACGGTTCTGGTAGTGGTTCC	GCTTCCTACCAAAACATAAAAGTGTCGGGCGGCCGTTGCACAACCAAAATTTCCTTCAAAACATTACCGGGTCACCAGCACCAATTTGAGAGACCTGTGC
ATPTb8 RNAi	TAATCTCGAGGGTACCGGGAGTACAGAAGGGCTACA	TAGATCTAGAGGATCCCGTGCACACCATGAGCTG
ATPTb8 qPCR	CAAGCCTTGCACACACTTTATG	CCGCAAAGAAGTACGCCAC

Primary antibodies used in this study		
**Primary antibody**	**Dilution**	**Reference (see main text reference list) or commercial supplier**
Anti-V5	1:2000	Invitrogen
Mouse Anti-mtHSP70	1:500	(K.A. Panigrahi, A. Zíková, R.A. Dalley, N. Acestor, Y. Ogata, A. Anupama, P.J. Myler, K.D. Stuart, Mol Cell Proteom 7:534-545, 2008, https://doi.org/10.1074/mcp.M700430-MCP200)
Rabbit anti-Mic10-1	1:1000	(31)
Rabbit anti-Mic60	1:1000	This study
Rabbit Anti-ATP β	1:2500	(K. Šubrtová, B. Panicucci, and A. Ziková, PLOS Pathog. 10(2): e1004660, 2015, https://doi10.1371/Journal.ppat.1004660)
Rabbit Anti-ATPTb2	1:1000	(K. Šubrtová, B. Panicucci, and A. Ziková, PLOS Pathog. 10(2): e1004660, 2015, https://doi10.1371/Journal.ppat.1004660)
Rabbit Anti-p18	1:1000	(O. Gahura, K. Šubrtová, H. Váchová, B. Panicucci, I.M. Fearnley, M.E. Harbour, J.E. Walker, A. Ziková, FEBS J 3:614-628, 2018, https://doi.org/10.1111/febs.14364)
Rabbit Anti-OSCP	1:1000	(C. Hierro-Yap, K. Šubrtová, O. Gahura, B. Panicucci, C. Dewar, C. Chinopoulos, A. Schnaufer, A. Ziková, J Biol Chem 100357, 2021, https://doi.org/10.1016/j.jbc.2021.100357)
